# Adverse drug reactions and correlations with drug–drug interactions: A retrospective study of reports from 2011 to 2020

**DOI:** 10.3389/fphar.2022.923939

**Published:** 2022-08-22

**Authors:** Huaqiao Jiang, Yanhua Lin, Weifang Ren, Zhonghong Fang, Yujuan Liu, Xiaofang Tan, Xiaoqun Lv, Ning Zhang

**Affiliations:** ^1^ Department of Pharmacy, Jinshan Hospital, Fudan University, Shanghai, China; ^2^ Department of Nursing, Jinshan Hospital, Fudan University, Shanghai, China

**Keywords:** adverse drug reactions, drug–drug interactions, causality, severity, preventability

## Abstract

**Introduction:** Adverse drug reactions (ADRs) represent a public health problem worldwide that deserves attention due to the impact on mortality, morbidity, and healthcare costs. Drug–drug interactions (DDIs) are an important contributor to ADRs. Most of the studies focused only on potential DDIs (pDDIs), while the detailed data are limited regarding the ADRs associated with actual DDIs.

**Methods:** This retrospective study evaluated ADRs reported between 2011 and 2020 in a tertiary hospital. The causality and severity of ADRs were evaluated through the Naranjo Algorithm and Hartwig’s scale, respectively. Preventability classification was based on the modified Schoumock and Thornton scale. For ADRs with at least two suspected drugs, pDDIs were identified according to the Lexi-Interact. We further checked whether the ADR description in the reports corresponded to the clinical consequences of the pDDIs.

**Results:** A total of 1,803 ADRs were reported, of which 36.77% ADRs were classified as mild, 43.26% as moderate, and 19.97% as severe. The assessment of causality showed that the distributions of definite, probable, and possible categories were 0.33%, 58.68%, and 40.99%, respectively. A total of 53.97% of ADRs were identified as preventable ADRs, while 46.03% were recognized as unpreventable. The severity of ADRs was significantly correlated with age, the number of suspected drugs and preventability. Antimicrobial agents were the most common implicated pharmacological group, and the most frequently affected system was the gastrointestinal system. Considering individual drugs, aspirin was the most frequently reported drug. Among 573 ADRs with at least two suspected drugs, 105 ADRs were caused by actual DDIs, of which only 59 and 6 ADRs were caused by actual DDIs in category D and X, respectively. The most frequent drugs involved in actual DDIs of category D were aspirin and heparin, with the majority of ADRs being gastrointestinal bleeding.

**Conclusion:** This study analyzed the pattern of ADRs in detail and obtained clinical evidence about ADRs associated with actual DDIs. These findings may be useful to compare patterns between different centers and to design preventive strategies for ADRs. Continuous education and training should be provided for physicians regarding the knowledge and recognition of ADRs associated with DDIs.

## Introduction

According to the World Health Organization (WHO), an adverse drug reaction (ADR) is an unintended and noxious response that is detected in patients after the use of drugs for the prophylaxis, diagnosis or treatment of a disease at doses normally used (Edwards and Aronson, 2000). ADRs, as a major threat in the healthcare system, contribute significantly to mortality, morbidity, extended hospital stays, and increased healthcare costs (Khan, 2013; Angamo et al., 2016). A meta-analysis showed that the percentage of ADR-induced admissions in patients over 60 years old was accurately estimated to be 8.7% (Oscanoa et al., 2017). To minimize the consequences of ADRs, it is necessary to study ADRs in terms of their early identification and prevention and to motivate healthcare professionals to report ADRs (Arulappen et al., 2018).

According to a WHO report, 60% of ADRs are preventable (Lau et al., 2003). Drug–drug interactions (DDIs) are an important cause of preventable ADRs. The increasing number of patients with multimorbidity and the growing complexity of therapeutic agents have led to widespread polypharmacy, which could result in the rising numbers of potential DDIs (pDDIs), especially in elderly individuals (Obreli-Neto et al., 2012a; Scondotto et al., 2018). Although there are several databases available that could be used to evaluate pDDIs, the clinical relevance and actual clinical importance of majority pDDIs remain insufficiently characterized and underestimated (Roblek et al., 2015). Actual DDIs are identified on the basis of clinical evidence, such as laboratory test results or symptoms, consequently, the frequency of actual DDIs is much lower than that of pDDIs (Magro et al., 2012; Zheng et al., 2018). Over the past years, a substantial number of articles have been published about ADRs due to DDIs (Leone et al., 2010; Obreli-Neto et al., 2012a; Obreli Neto et al., 2012b; Kovacevic et al., 2019; Letinier et al., 2020; Magro et al., 2020). A 6-year retrospective study in Bengbu in China showed that among the ADRs reported between nervous system drugs in hospitalized patients, 12.14% of the ADRs were associated with potential and actual DDIs, and actual DDIs were present in 6.21% of all ADRs (Shi et al., 2014). However, the incidence of ADRs resulting from DDIs could not be accurately estimated primarily because of differences in study designs and populations (Mirosevic Skvrce et al., 2011).

In this context, the present study aimed to describe the distribution of ADRs, assess causality, preventability and severity of ADRs, and determine factors involved in the severity of ADRs in a tertiary hospital between 2011 and 2020. Additionally, we described and analyzed the most frequent drugs suspected to cause ADRs and the organ system classes affected by ADRs. Furthermore, we evaluated the pDDIs among the ADRs with more than one suspected drug, estimated the incidence of ADRs due to actual DDIs and characterized ADRs caused by actual DDIs.

## Materials and methods

### Data collection

In this retrospective single-center study, all the ADRs was collected from the National ADR Monitoring system in Jinshan Hospital of Fudan University, between 01 January 2011 and 31 December 2020. Jinshan Hospital is a tertiary general hospital with a 700-bed capacity in the Jinshan district of Shanghai. In 2020, there were 28,533 hospital admissions, and 1.28 million outpatient and emergency department visits. ADR reports were filled out according to a specific ADR report format and submitted in paper based or electronic way by healthcare professionals, including physicians, pharmacists, and nurses.

Once received, the reported ADRs were reviewed and evaluated by ADR surveillance unit of the pharmacy department. Only the reported ADRs followed the WHO definition (Edwards and Aronson, 2000) and without any uncertainty or mistakes were accepted after exclusion of duplicates and uploaded to ADR Monitoring system. A series of exclusion criteria were applied to ensure a robust data set for analysis. Exclusion criteria included the following: 1) ADRs with doubtful causality with Naranjo’s algorithm (Naranjo et al., 1981). 2) ADR forms with insufficient information 3) ADRs symptoms similar to the original disease.

**FIGURE 1:**
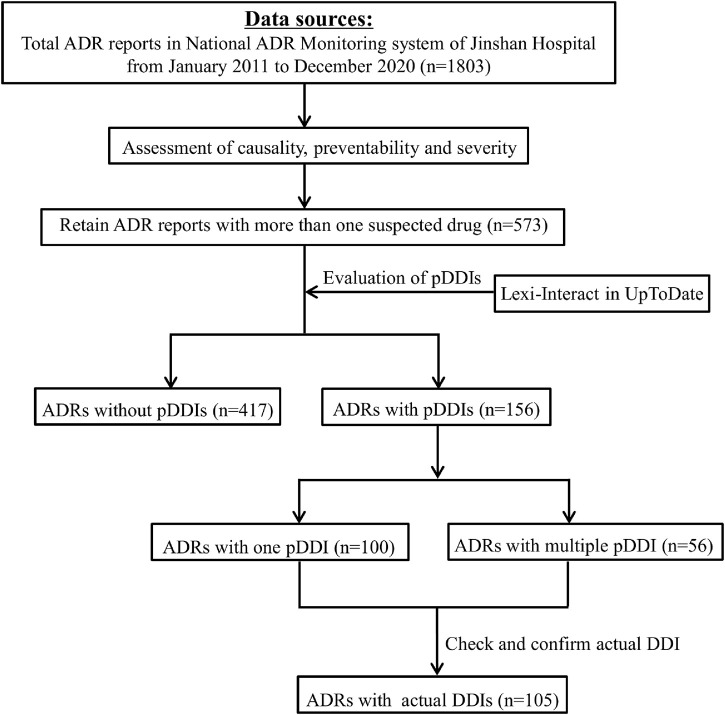
Flowchart depicting the study process.

The demographic and other information relevant to ADRs were documented, including gender, age, diagnosis, admission department, suspected drugs, concomitant medications, drug details, organ system involved in the ADR, the management and outcome of the ADRs, and the type of reporter. One report could describe one or more ADRs. The incriminated drugs were classified by pharmacological group according to the WHO Anatomical Therapeutic Chemical Classification (ATC). The involved system organ classes were determined according to WHO Adverse Reaction Terminologies (WHO-ART). Two investigators cross checked the data for accuracy. Flowchart depicting the study process was shown in Figure 1.

### Causality, preventability, and severity assessment

Each ADR was further evaluated for various parameters, such as causality, severity and preventability, using previously validated and recognized approaches. The assessment of causality was performed using the Naranjo Algorithm, which consists of 10 individually scored criteria. ADRs were categorized as possible ADRs (1–4), probable ADRs (5–8) or definite ADRs (≥9) based on the total score (Naranjo et al., 1981). Severity classification was based on Hartwig’s scale, which showed the criteria and matched levels used for ADR severity assessment. ADRs were considered as severe if they resulted in one of the following outcomes: the requirement for intensive medical care, permanent harm to the patient, or the death of the patient (Hartwig et al., 1992). The preventability of ADRs was assessed by the modified Schoumock and Thornton scale and classified into definitely preventable, probably preventable and not preventable reactions (Schumock and Thornton, 1992). In our study, both definitely and probably preventable ADRs were considered as one category of preventable reactions.

### Evaluation of potential drug–drug interactions

For ADRs caused by two or more suspected drugs, pDDIs were identified by the software Lexi-Interact in UpToDate. The evaluation results of pDDIs were classified into five levels of risk as no known interaction (A), no action needed (B), monitor therapy (C), consider therapy modification (D), and avoid combination (X). We further verified whether the clinical consequences of pDDIs corresponded to the description of the ADR in the report, and if consistent, the pDDI was considered the actual DDI. Two clinical pharmacists independently assessed the probability, severity and preventability of ADRs as well as the consistency between ADRs and pDDIs. Any discrepancies were resolved by discussion.

### Statistical analysis

Descriptive statistics were applied to describe the population as well as the clinical characteristics of ADRs and pDDIs. The categorical data were presented as numbers and proportions. Sankey diagrams of severity in preventable and unpreventable ADRs were plotted with the R package alluvial. The Mann–Whitney *U* test was used to evaluate the correlation between gender and the severity of ADRs. Spearman’s rank tests were performed to determine the association of age, the number of suspected drugs and the category of preventability with the severity of ADRs. The Kruskal–Wallis *H* test was performed to evaluate the correlation between the route of administration and the severity of ADRs. Statistical analysis was performed using IBM SPSS Statistics version 25. A *p*-value < 0.05 was considered statistically significant.

## Results

### Department and reporter distribution of adverse drug reactions

**FIGURE 2:**
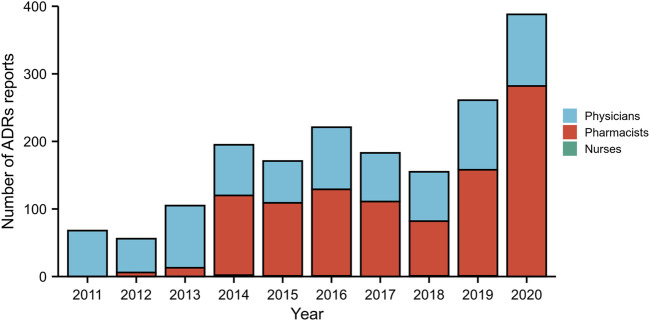
The total number of adverse drug reaction (ADR) reports and the distribution of reporters from different occupations by year during 2011–2020.

**FIGURE 3:**
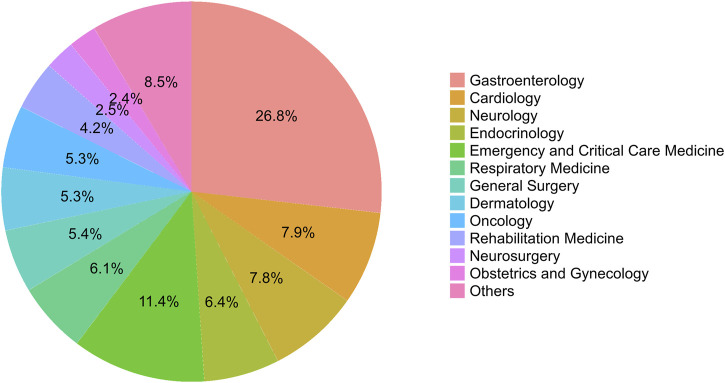
The percentage of adverse drug reactions (ADRs) from different clinical departments.

From January 2011 to December 2020, a total of 1,803 ADRs were reported by healthcare professionals in our hospital, although the number of ADRs reported was relatively small between 2011 and 2013. During this 10-year period, pharmacists contributed 55.69% of all ADR reports, followed by physicians (43.98%). The frequency of ADRs reported by nurses was low, accounting for only 0.33%. The annual number of reports was no more than 221 during 2011–2018, however, this number subsequently increased significantly over the next 2 years, reaching 388 in 2020 (Figure 2). A small proportion of ADRs were reported by pharmacists between 2011 and 2013, however, since 2014, more than half of ADR reports have been submitted by pharmacists. Detailed data by the year and distribution of reporters were shown in Figure 2. In our study, the highest percentage of ADRs was collected from the gastroenterology department (26.8%), followed by the departments of emergency and critical care medicine (11.4%), cardiology department (7.9%), and neurology department (7.8%) (Figure 3). The proportions of ADRs collected from clinical departments were presented in Figure 3.

### Causality, preventability, and severity assessment of adverse drug reactions

**FIGURE 4:**
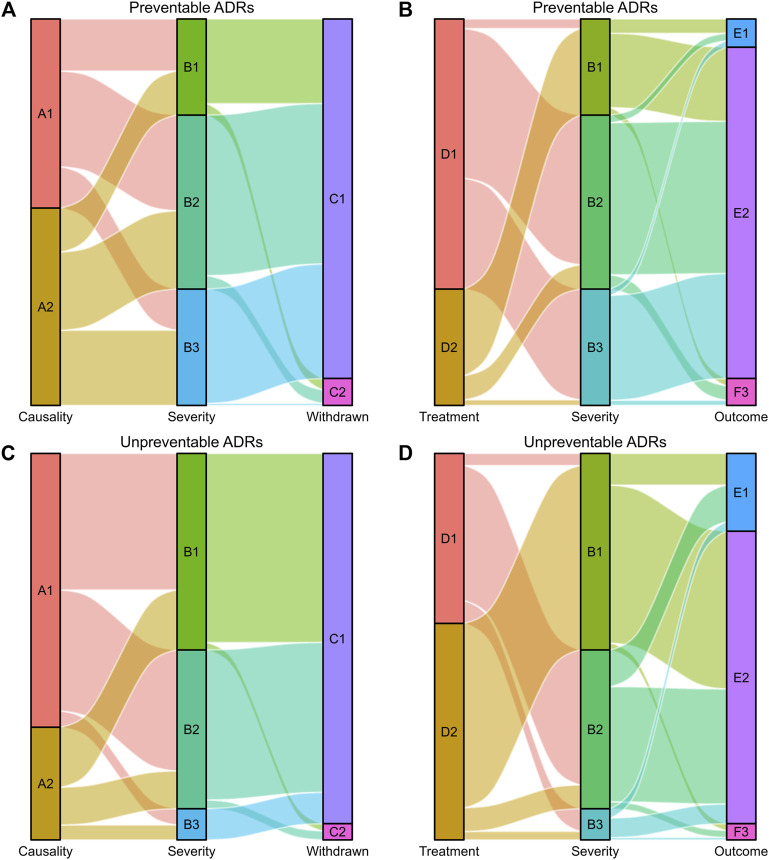
Sankey diagram of severity in preventable and unpreventable ADRs. **(A,B)** The causality assessment, fate of the suspected drug, treatment given and outcome of ADRs matched with ADR severity in preventable ADRs. **(C,D)** The causality assessment, fate of the suspected drug, treatment given and outcome of ADRs matched with ADR severity in unpreventable ADRs. The causality assessment of ADRs (A1 and A2), A1: Definite/Probable, A2: Possible. ADR severity (B1, B2, and B3), B1: Mild, B2: Moderate, B3: Severe. Fate of the suspected drug (C1 and C2), C1: Drug withdrawn, C2: Dose altered/No change. Treatment given (D1 and D2), D1: Treatment given, D2: No treatment. Outcome of ADRs (E1, E2, and E3), E1: Recovered, E2: Improved, E3: Continuing/Unclear.

ADRs were further analyzed for causality, preventability and severity, as shown in Table 1. The assessment of causality according to the Naranjo Algorithm showed that the numbers of definite, probable and possible ADRs were 6 (0.33%), 1,058 (58.68%), and 739 (40.99%), respectively. According to Hartwig’s Severity Assessment Scale, 663 (36.77%) ADRs were classified as mild, 780 (43.26%) as moderate, and 360 (19.97%) as severe. The evaluation of the preventability of ADRs using the modified Schumock and Thornton criteria revealed that 973 (53.97%) ADRs were identified as preventable ADRs, including 93 as definitely preventable and 880 as probably preventable, while 830 (46.03%) ADRs were recognized as unpreventable. Symptomatic or specific treatment was given for 1,045 (57.96%) ADRs. According to the records of ADR reports, the majority of ADRs (81.09%) had improved, 238 (13.20%) patients had recovered from their ADRs, and 103 (5.71%) ADRs continued or their status was unclear. Suspected drugs were withdrawn in 1,700 (94.29%) ADR reports, but an altered dose or no change in therapy was observed in 103 (5.71%) reports. The visual design follows the principle of the Sankey diagram, which links the ADR characteristics by lines and signifies the quantities *via* line width, stratified by preventability (Figure 4).

**TABLE 1:** Assessment and pattern of adverse drug reactions.

Variable	Number of ADRs (%)
Causality assessment	
Definite/probable	1,064 (59.01)
Possible	739 (40.99)
Severity	
Mild	663 (36.77)
Moderate	780 (43.26)
Severe	360 (19.97)
Preventability	
Definitely/probably preventable	973 (53.97)
Unpreventable	830 (46.03)
Treatment given	
Yes	1,045 (57.96)
No	758 (42.04)
Outcome of ADRs	
Recovered	238 (13.20)
Improved	1,462 (81.09)
Continuing/unclear	103 (5.71)
Fate of the suspected drug	
Drug withdrawn	1,700 (94.29)
Dose altered/rechallenge	103 (5.71)

### Characteristics of adverse drug reactions according to severity assessment

The characteristics of ADRs according to severity assessment were shown in Table 2. A total of 1,803 ADRs were identified among 1,779 patients. Multiple ADRs in the same patient may be identified with different severity scale, so Table 2 depicted the distribution of mild, moderate, and severe reactions between different gender and age based on ADRs rather than patients. Concerning patient gender and ADRs, 46.87% males and 53.13% females experienced ADRs over the past 10 years. The proportion of mild ADRs was higher in females (60.33%) than in males (39.67%), however, the ratio of males to females was approximately 1:1 among those experiencing moderate and severe ADRs. The Mann–Whitney *U* test revealed significant differences in the mild, moderate, and severe ADR distributions between the males and females.

**TABLE 2:** Comparison of mild, moderate, and severe reactions.

Characteristics	Total, *n* (%)	Mild, *n* (%)	Moderate, *n* (%)	Severe, *n* (%)	*p*-value	*R*
Gender						
Male	845 (46.87)	263 (39.67)	399 (51.15)	183 (50.83)	<0.001^a^	—
Female	958 (53.13)	400 (60.33)	381 (48.85)	177 (49.17)		
Age (years)						
<18	85 (4.71)	18 (2.71)	66 (8.46)	1 (0.28)	<0.001^b^	0.167
18–40	253 (14.03)	130 (19.61)	104 (13.33)	19 (5.28)		
41–64	692 (38.38)	286 (43.14)	269 (34.49)	137 (38.06)		
≥65	773 (42.87)	229 (34.54)	341 (43.72)	203 (56.39)		
Number of suspected drugs						
1	1,221 (67.72)	499 (75.26)	512 (65.64)	210 (58.33)	<0.001^b^	0.136
2	439 (24.35)	126 (19.00)	202 (25.90)	111 (30.83)		
≥3	143 (7.93)	38 (5.73)	66 (8.46)	39 (10.83)		
Preventability						
Unpreventable	830 (46.03)	422 (63.65)	341 (43.72)	67 (18.61)	<0.001^b^	0.299
Probably preventable	880 (48.81)	210 (31.67)	392 (50.26)	278 (77.22)		
Definitely preventable	93 (5.16)	31 (4.68)	47 (6.03)	15 (4.17)		
Route of administration						
Oral	1,019 (56.52)	340 (51.28)	409 (52.44)	270 (75.00)	<0.001^c^	
Intravenous	683 (37.88)	281 (42.38)	321 (41.15)	81 (22.50)		
Others	101 (5.60)	42 (6.33)	50 (6.41)	9 (2.50)		

a Mann–Whitney *U* test.

b Spearman.

c Kruskal–Wallis H.

The *R* value represents Spearman’s correlation coefficient.

As shown in Table 2, the percentage of ADRs was highest among elderly individuals over 65 years of age (42.87%), followed by the 41–64-year (38.38%) and 18–40-year (14.03%) age groups. The minimum number of ADRs was observed in the age group under 18 years (4.71%). More than half of severe ADRs occurred in elderly individuals over 65 years of age. The majority of ADRs (67.72%) were identified with only one suspected drug, followed by 24.35% with two suspected drugs, and only 143 (7.93%) ADRs were found with ≥3 suspected drugs. According to Hartwig’s scale, 46.03% of ADRs were classified as unpreventable ADRs, 48.81% as probably preventable ADRs and 5.16% as definitely preventable ADRs. The percentage of unpreventable ADRs significantly decreased with ADR severity (mild 63.65% vs. moderate 43.72% vs. severe 18.61%). The statistical results revealed significant positive correlations of ADR severity with age (Spearman’s *R* = 0.167, *p* < 0.001), the number of suspected drugs (Spearman’s *R* = 0.136, *p* < 0.001) and ADR preventability (Spearman’s *R* = 0.299, *p* < 0.001).

The route of administration was classified according to the first suspected drug in the ADR reports. More than half of ADRs were associated with oral medicines regardless of their severity rating. Unexpectedly, the proportion of ADRs associated with intravenous drugs gradually decreased with increasing severity. The Kruskal–Wallis *H* test was further carried out and showed a significant association between the route of administration and the severity of ADRs (Table 2).

### Drugs involved in adverse drug reactions and effects on organ systems

The pharmacological groups implicated in the ADRs were summarized in Table 3. Systemic antimicrobial agents were the most commonly implicated drugs (22.75%), with 14.53% and 39.32% of their associated ADRs being classified as severe and preventable ADRs, respectively. Cardiovascular agents were the second most frequently reported class of drugs responsible for ADRs (12.41%), followed by medications for the alimentary tract and metabolism (12.06%). Drugs acting on the blood and blood-forming organs represented 11.75% of the reports (32.45% severe and 83.11% preventable ADRs). Drugs acting on the musculoskeletal system were implicated in 10.77% of the ADRs (39.71% severe and 71.48% preventable ADRs). Traditional Chinese medicines were implicated in 10.07% of the reports (18.53% severe ADRs and 54.44% preventable ADRs).

**TABLE 3:** Pharmacology groups according to the WHO-ATC code and their pattern in ADRs.

Pharmacology groups	Number of patients	ADR frequency (%)	Number of severe ADRs (%)	Number of preventable ADRs (%)
Alimentary tract and metabolism	256	310 (12.06)	65 (20.97)	51 (16.45)
Blood and blood-forming organs	230	302 (11.75)	98 (32.45)	251 (83.11)
Cardiovascular system	281	319 (12.41)	85 (26.65)	208 (65.20)
Dermatologicals	6	7 (0.27)	1 (14.29)	6 (85.71)
Genito urinary system and sex hormones	9	9 (0.35)	1 (11.11)	3 (33.33)
Systemic hormonal preparations, excl. sex hormones, and insulins	72	76 (2.96)	15 (19.74)	13 (17.11)
Anti-infectives for systemic use	503	585 (22.75)	85 (14.53)	230 (39.32)
Antineoplastic and immunomodulating agents	129	192 (7.47)	24 (12.50)	161 (83.85)
Musculo-skeletal system	253	277 (10.77)	110 (39.71)	198 (71.48)
Nervous system	153	163 (6.34)	32 (19.63)	86 (52.76)
Antiparasitic products, insecticides, and repellents	4	4 (0.16)	0	2 (50.00)
Respiratory system	39	42 (1.63)	5 (11.90)	20 (47.62)
Sensory organs	4	4 (0.16)	0	0
Traditional chinese medicine	240	259 (10.07)	48 (18.53)	141 (54.44)
Others	22	22 (0.86)	0	5 (22.73%)

a Each ADR may have multiple suspected drugs, therefore the total number of incriminated drugs exceeds the ADRs.

WHO-ART, WHO Adverse Reaction Terminologies; ADR, adverse drug reaction.

The frequency of commonly prescribed drugs among total and severe ADRs was shown in Table 4. When individual drugs were considered, aspirin was responsible for a maximum number of both total and severe ADRs, far more than any other drugs. Among the total ADRs, levofloxacin (82) was the second most frequent causative drug, followed by compound pseudoephedrine hydrochloride (65) and clopidogrel (48). In addition to aspirin, the drugs most frequently involved in severe ADRs were clopidogrel (17), levofloxacin (16), compound pseudoephedrine hydrochloride (14), and diclofenac sodium (13).

**TABLE 4:** Top 10 incriminated drugs in total and severe ADRs based on frequency.

Ranking	Total ADRs		Severe ADRs	
	Drugs	Frequency (*n*)	Drugs	Frequency (*n*)
1	^a^Aspirin	134	^a^Aspirin	52
2	Levofloxacin	82	Clopidogrel	17
3	^a^Compound pseudoephedrine hydrochloride	65	Levofloxacin	16
4	Clopidogrel	48	^a^Compound pseudoephedrine hydrochloride	14
5	Moxifloxacin	47	^a^Diclofenac sodium	13
6	^a^Diclofenac sodium	42	^a^Paracetamol, aminophenazone, caffeine, and chlorphenamine maleate	11
7	Metformin	40	^a^Analgin	10
8	Azithromycin	39	Cefoperazone sodium and sulbactam sodium, warfarin, lansoprazole, compound reserpine	8
9	Rosuvastatin	34	Valsartan, metformin, compound irbesartan, and hydrochlorothiazide	7
10	Cefuroxime	33	^a^Ibuprofen, ^a^compound ibuprofen and codeine, ^a^compound paracetamol, caffeine and aspirin, rosuvastatin	6

a These drugs belong to the category of non-steroidal anti-inflammatory drugs (NSAIDs).

ADR, adverse drug reaction.

Upon a review of the outcomes of ADRs, the most frequently affected system was the gastrointestinal system (30.83%), with the clinical symptoms of nausea, vomiting, abdominal pain, diarrhea, abdominal distention, and so on. In addition, the commonly reported reactions were skin and appendage disorders (22.44%) and liver and biliary system disorders (14.19%). A more detailed description was presented in Table 5.

**TABLE 5:** Organs or systems involved in ADRs according to WHO classification.

Organs/systems	Clinical manifestations/symptoms	Frequency (%)
Skin and appendages disorders	Itching, urticaria, rash, maculopapular rash, erythema, etc.,	639 (22.44)
Musculo-skeletal system disorders	Myasthenia, myalgia, muscle bleeding, arthralgia, lower limb spasm, osteoporosis	26 (0.91)
Central and peripheral nervous system disorders	Dizziness, headache, peripheral neuropathy, coma, grand mal seizure, manic-depressive psychosis, etc.,	186 (6.53)
Autonomic nervous system disorders	Red flush, erythromelalgia	3 (0.11)
Vision disorders	Ocular abnormality, conjunctival hemorrhage, ocular pain, blurred vision	6 (0.21)
Hearing and vestibular disorders	Tinnitus	5 (0.18)
Special senses other, disorders	Taste perversion	1 (0.04)
Psychiatric disorders	Circulatory psychotic reactions, insomnia, manic reactions, sleep disorders, neurosis, etc.,	28 (0.98)
Gastro-intestinal system disorders	Nausea, vomiting, abdominal pain, gastrointestinal bleeding, abdominal distention, flatulence, black feces, diarrhea, hematemesis, etc.,	878 (30.83)
Liver and biliary system disorders	Abnormal liver function, jaundice, elevated liver enzymes, cholestatic hepatitis, biliary cirrhosis	404 (14.19)
Metabolic and nutritional disorders	Electrolyte abnormality, hyperuricemia, increased blood lactic acid, hypokalemia; hyponatremia, hyperkalemia, hypoglycemia, hyperglycemia, etc.,	52 (1.83)
Endocrine disorders	Male breast pain, non-specific endocrine disease, thyroiditis, hyperparathyroidism	5 (0.18)
Cardiovascular disorders, general	Hypotension, hypertension	64 (2.25)
Heart rate and rhythm disorders	Palpitations, tachycardia, bradycardia, cardiac arrest, arrhythmias, atrioventricular block	35 (1.23)
Respiratory system disorders	Dyspnea, asthma, cough	34 (1.19)
Red blood cell disorders	Anemia	3 (0.11)
White cell and respiratory disorders	Leukopenia, leukopenia, granulocytopenia, and granulocytopenia	8 (0.28)
Platelet, bleeding, and clotting disorders	Bone marrow suppression, thrombocytopenia, coagulopathy, hematemesis, etc.,	119 (4.18)
Urinary system disorders	Hematuria, abnormal renal function, urinary retention	54 (1.90)
Reproductive disorders, female	Genital itching, breast enlargement, menstrual disorders	3 (0.11)
Body as a whole—general disorders	Fatigue, allergic reactions, chills	220 (7.72)
Application site disorders	Phlebitis, skin necrosis	70 (2.46)
Resistance mechanism disorders	Decreased IgG4, systemic lupus erythematosus syndrome	5 (0.18)
Total^a^		2,848 (100%)

a Some ADRs with multiple system or organ disorders.

WHO, World Health Organization; ADR, adverse drug reaction.

### Adverse drug reactions caused by drug–drug interactions

pDDIs were evaluated in 573 of 1,803 ADR reports (31.78%) involving more than one suspected drug. 156 ADRs were identified with pDDIs of category C, D, and X, of which 100 ADRs were identified with only one pDDI and 56 ADRs with multiple pDDIs. Table 6 showed that 208 pDDIs of category C were identified in 112 ADRs, 74 pDDIs of category D in 58 ADRs, and 11 pDDIs of category X in 10 ADRs. Furthermore, we checked whether the reported ADRs were consistent with the potential clinical consequences of pDDIs. The results showed 105 ADRs were caused by actual DDIs, accounting for 18.32% of the ADR reports with more than one suspected drug. Among them, 59 and 6 ADRs were caused by actual DDIs in the category D and X, respectively.

**TABLE 6:** Distribution of the potential drug–drug interactions with category C, D, and X in ADRs.

Risk rating	Type of drug-drug interaction	ADRs (*n*)	pDDIs (*n*)
C	Monitor therapy	112	208
D	Consider therapy modification	58	74
X	Avoid combination	10	11

ADR, adverse drug reaction; pDDI, potential drug–drug interaction.

Tables 7, 8 summarized the ADRs caused by actual DDIs belonging to category X and D, respectively. Potassium chloride and promethazine were the drug–drug combination most involved in ADRs caused by actual DDIs in category X, with severe and adverse clinical consequences to the gastrointestinal system. The most frequent drugs involved in actual DDIs of category D were aspirin (*n* = 34) and heparin (*n* = 26), and the great majority of ADRs caused by DDIs were associated with gastrointestinal bleeding. Aspirin/heparin (*n* = 10) and heparin/clopidogrel (*n* = 10), followed by aspirin/warfarin (*n* = 6) and aspirin/ibuprofen (*n* = 5), were the drug–drug combinations most involved in ADRs caused by DDIs of category D.

**TABLE 7:** ADRs caused by actual drug–drug interactions belonging to category X.

Drug pairs	*n*	Reliability of pDDIs	Potential clinical consequences	Reported ADRs	Severity of ADRs
Diclofenac-indomethacin	1	Fair	Increased the risk of gastrointestinal toxicity	Gastrointestinal bleeding, melena	Moderate
Dexamethasone-desmopressin	1	Fair	Increased the risk of hyponatremia	Electrolyte abnormalities, edema	Severe
Potassium chloride- chlorphenamine	1	Fair	Enhanced the ulcerogenic effect of potassium chloride	Gastrointestinal bleeding	Severe
Potassium chloride- Promethazine	3	Fair	Enhanced the ulcerogenic effect of potassium chloride	Gastrointestinal bleeding (1), abdominal pain and anorexia (1), gastritis, and abdominal distension (1)	Severe-3

ADR, adverse drug reaction; pDDI, potential drug–drug interaction.

**TABLE 8:** ADRs caused by actual drug–drug interactions belonging to category D.

Drug pairs	*n*	Reliability of pDDIs	Potential clinical consequences	Reported ADRs	Severity of ADRs
Aspirin-loxoprofen	2	Good	Increased risk of bleeding	Gastrointestinal bleeding (2)	Severe (2)
Aspirin-warfarin	6	Excellent	Enhanced anticoagulant effect	Gastrointestinal bleeding (4), hematuria (1) and gingival bleeding (1)	Severe (2), Moderate (4)
Aspirin-heparin	10	Good	Enhanced anticoagulant effect	Gastrointestinal bleeding (3), hematuria and melena (2), hematuria (1), epistaxis (1), non-specific hemorrhage (1), coagulopathy (1), and hemorrhagic dermatitis (1)	Severe (4), Moderate (4), Mild (2)
Aspirin-ginkgo	4	Fair	Enhanced anticoagulant effect	Gastrointestinal bleeding (2), gingival and gastrointestinal bleeding (1), hematuria and melena (1)	Severe (4)
Aspirin-diclofenac	3	Good	Increased risk of bleeding	Gastrointestinal bleeding (2), hematemesis (1)	Moderate (2),Severe (1)
Aspirin-ibuprofen	5	Good	Increased risk of bleeding	Gastrointestinal bleeding (5)	Severe (3), Moderate (2)
Aspirin-celecoxib	1	Good	Enhanced adverse effect	Gastrointestinal bleeding	Severe
Aspirin-ticagrelor	1	Fair	Enhanced antiplatelet effect	Melena	Moderate
Aspirin-propyphenazone	1	Good	Increased risk of bleeding	Gastrointestinal bleeding	Moderate
Aspirin-rivaroxaban	1	Fair	Increased risk of bleeding	Gastrointestinal bleeding	Moderate
Heparin-clopidogrel	10	Good	Enhanced anticoagulant effect	Gastrointestinal bleeding (3), hematuria and melena (2), hematuria (1), epistaxis (1), non-specific hemorrhage (1), cerebral hemorrhage (1), muscle hemorrhage (1)	Severe (4), Moderate (5), Mild (1)
Heparin-dipyridamole	2	Good	Enhanced anticoagulant effect	Gingival and gastrointestinal bleeding (1), hematuria and melena (1)	Severe (2)
Heparin-tirofiban	4	Good	Enhanced anticoagulant effect	Hematuria and melena (1), non-specific hemorrhage (1), gastrointestinal bleeding (1), thrombocytopenia (1)	Severe (2), Moderate (1), Mild (1)
Docetaxel-carboplatin	2	Fair	Increased myelosuppressive effect	Thrombocytopenia (1) and leukopenia (1)	Moderate (2)
Docetaxel-epirubicin	1	Excellent	Enhanced adverse effect	Myelosuppression and fatigue	Moderate
Docetaxel-cisplatin	1	Fair	Increased myelosuppressive effect	Myelosuppression	Moderate
Digoxin-amiodarone	1	Excellent	Increased serum concentration of digoxin	Atrioventricular block and bradycardia	Severe
Dihydrocodeine-tizanidine	1	Fair	Enhanced CNS depressant effect	Somnolence	Mild
Rivaroxaban-clopidogrel	1	Fair	Increased risk of bleeding	Gastrointestinal bleeding	Moderate
Glimepiride-acarbose	1	Fair	Enhanced hypoglycemic effect	Hypoglycemia	Mild
Amikacin-vancomycin	1	Fair	Enhanced nephrotoxic effect of aminoglycosides	Abnormal renal function	Moderate

ADR, adverse drug reaction; CNS, central nervous system.

## Discussion

In this study, physicians and pharmacists were the groups that reported the great majority of ADRs, and the frequency of ADRs reported by nursing staff was low, which may be due to their extensive workload in everyday practice, inattention and unawareness toward ADR reporting or worry about legal implications (Singh et al., 2017). The reporter distribution of ADRs varies widely in different studies because of differences in healthcare structures as well as the awareness and motivation of healthcare professionals. The number of ADRs was relatively small, especially for ADRs reported by pharmacists between 2011 and 2013, indicating underreporting in pharmacovigilance. The key to improving ADR reporting rates is adequate pharmacovigilance education and training for healthcare professionals (Barzaga Arencibia et al., 2012).

In the present study, we analyzed the pattern of ADRs based on the causality, severity, and preventability in our hospital, all of which vary among different hospitals due to differences in the population characteristics and hospital specialties. Naranjo’s causality assessment showed that only 0.33% of reports were definite because of limited use of dechallenge and rechallenge processes for ethical reasons as well as the retrospective study design without the ability to assess the ADR completely. The suspected drugs were withdrawn among 94.29% of ADRs, and for the remaining 5.71% of ADRs, the suspected drug doses were altered or rechallenge processes were initiated. In this study, 19.97% of ADRs were classified as severe. Severe ADRs, as major concerns for public health, are a contributing factor of hospitalizations and morbidity (Rottenkolber et al., 2011; Marques et al., 2014). The analysis indicated a preventability rate of 53.97% among ADRs, comparable with the results of studies conducted in Romania and Jordan showing that 41% and 44.7% of ADRs were preventable, respectively (Farcas et al., 2014; Al Damen and Basheti, 2019). However, the data from a study showed lower preventability for ADRs (12%) compared with our finding (Dequito et al., 2011). As described in previous studies, insufficient monitoring, inappropriate dosing, and DDIs were the most frequent factors involved in ADR preventability (Farcas et al., 2014; Al Damen and Basheti, 2019). Incriminated drugs were withdrawn in 94.29% of the reports, which is in line with a previous study in a psychiatric department of a tertiary care teaching hospital in India (Patel et al., 2015). The high proportion of withdrawal may be due to the reporting nature of ADRs that troublesome ADRs are more likely to be detected.

There may be significant difference between male and female regarding the ADR prevalence due to factors such as body mass index, fat composition, hormonal effects, drug susceptibility, or genetic differences in the levels of enzymes (Haile et al., 2013; Rukmangathen et al., 2020). However, we demonstrated that females had only slightly higher incidence of ADRs than males in the present study. The frequency of ADRs increased with age, with the highest prevalence of ADRs in elderly individuals over 65 years (42.87%), followed by individuals 41–64 years of age (38.38%), which is in concordance with the findings of a previous study (Shepherd et al., 2012). Older patients are particularly vulnerable to ADRs owing to the multiple-drug regimens used for chronic diseases and physiological changes in this population, such as reduced gastrointestinal motility and gastric blood flow, impaired repair mechanisms, and lower mucosal protection (Marusic et al., 2014). A systematic review of ADRs in elderly individuals revealed that comorbid complexity was positively associated with ADR occurrence (Alhawassi et al., 2014). In the present study, there were statistically significant differences in the incidence of severe ADRs in the different gender and age groups, and polypharmacy increased the proportion of severe ADRs.

Anti-infectives for systemic use were the most common pharmacological group, accounting for 22.75% of total ADRs in our study, which is in line with previous studies (Haile et al., 2013; Marques et al., 2014). The excessive use of antibiotics may be responsible for the increased risk of ADRs. Cardiovascular system agents (12.41%) were the second most frequently incriminated pharmacological class of ADRs in our study, among them, 65.20% were preventable ADRs. A systematic review showed that cardiovascular medicines were commonly associated with preventable drug-related admissions (Howard et al., 2007). In another study, cardiovascular agents were identified as the second most frequently responsible drugs linked to preventable ADRs (Farcas et al., 2014).

The system most frequently affected by ADRs in this study was the gastrointestinal system, accounting for 30.83%, probably due to more than half of the suspected drugs being administered orally. This was followed by skin and appendage disorders (22.44%). This observation is consistent with the findings of a prospective observational study of hospitalized pediatric patients, which reported gastrointestinal system disorders (51.56%) and skin and appendage disorders (18.75%) as the most frequent manifestations of ADRs (Kurian et al., 2016).

As DDIs are usually predictable and manageable, ADRs caused by DDIs may be prevented by monitoring the patient closely or replacing the responsible drugs with other medications. To reduce the risk of DDIs and improve patient safety, it is essential that healthcare professionals regularly review the medication regimens, recognize potentially interacting drug pairs, and withdraw unnecessary drugs (Magro et al., 2020). A prospective study showed that the number of patients with pDDIs and actual DDIs decreased by 18% and 43%, respectively, with an intervention based on a computerized clinical decision support system containing information on drug combinations (Bertsche et al., 2010). However, reporters less frequently recognize actual DDIs due to the limited availability of DDI databases or alerting drug-interaction systems (Mirosevic Skvrce et al., 2011). Therefore, it is important to increase the knowledge of pharmacovigilance through the additional education of healthcare providers.

In a previous study, we investigated the prevalence of pDDIs and their association with characteristics in outpatient prescriptions (Ren et al., 2020). However, to assess the clinical impact of DDIs on public health, only ADRs associated with DDIs should be considered. In our study, 105 ADR reports were induced by actual DDIs, accounting for 18.32% of the ADR reports with more than one suspected drug. This percentage was close to the proportion reported by Magro et al. (2020). According to the online version of DRUGDEX^®^ system, they verified DDI among serious ADRs containing at least two suspected or concomitant drugs in the National Pharmacovigilance database from Veneto Region, and identified 17.4% ADR reports associated with a DDI. However, the results of another study performed in an Italian spontaneous reporting database showed that regarding patients treated with at least two drugs, 6.5% of ADR reports was associated with a DDI using the DRUGDEX^®^ system (Leone et al., 2010). Similarly, a prospective cohort study conducted in the primary public health system of the Ourinhos microregion in Brazil revealed that the incidence of DDI-related ADRs was 6% in elderly outpatients using DDI-checker programs (DrugDigest^®^, Drugs^®^, Micromedex^®^, and Medscape^®^) (Obreli-Neto et al., 2012b).

In the present study, aspirin and heparin were the drugs most frequently associated with actual DDIs of category D, with symptom of gastrointestinal bleeding. Similarly, a prospective observational study conducted in the cardiology unit of an Indian hospital showed that heparin and aspirin were the most common drugs responsible for DDIs, and bleeding was the most frequent clinical consequence (Mateti et al., 2011). Furthermore, aspirin, which is widely used for the prevention of vascular events, was reported to increase the baseline risk of gastrointestinal bleeding by approximately 60% among older persons aged over 70 years in a randomized controlled trial (Mahady et al., 2021).

Although the study had important findings regarding the pattern of ADRs and the role of actual DDIs in ADRs over the past decade along with a large sample size, several limitations should be taken into consideration. First, as a retrospective study, data were collected from the clinical records of ADRs always with incomplete information, such as information on concomitant drugs, comorbidities, lifestyle, diet, and so on. Prospective studies will be carried out to clarify and reduce this limitation in the future. Second, this study was conducted at a single institution, limiting the generalizability of its findings due to the differences in population characteristics and prescribing patterns. Last, the single source of the DDI screening database used in this study may hinder the identification of DDIs because consistent criteria for DDI identification and assessment are currently lacking.

## Conclusion

This study of ADR data collected over 10 years revealed that almost all ADRs were reported by pharmacists and physicians in our hospital, and the severity of ADRs was significantly correlated with age, the number of suspected drugs and preventability. Systemic antimicrobial agents were the most frequently incriminated pharmacological group, and aspirin was responsible for the largest proportion of total and severe ADRs. The gastrointestinal system was the system most frequently affected by ADRs. As observed in this study, aspirin and heparin were the most common drugs in actual DDIs of category D, resulting in gastrointestinal bleeding.

Active pharmacovigilance programs are important to accurately identify and assess ADRs in the clinical setting, further minimize drug-induced harm and improve the quality of patient care. Our findings obtained clinical evidence about ADRs associated with actual DDIs in our hospital. It will be necessary to make clinicians aware of the possibility of DDI-related ADRs and achieve a clear understanding of drug pairs resulting in DDI-related ADRs, in order to guide the prescribing practices and minimize the harms from actual DDIs. Moreover, rigorous prescription and frequent monitoring of drug therapy are essential for reducing the risk of ADRs.

## Data Availability

The raw data supporting the conclusion of this article will be made available by the authors, without undue reservation.
